# In Limbo: Time Perspective and Memory Deficit Among Female Survivors of Sexual Abuse

**DOI:** 10.3389/fpsyg.2019.00912

**Published:** 2019-04-24

**Authors:** Angi Jacobs-Kayam, Rachel Lev-Wiesel

**Affiliations:** The Emili Sagol Creative Arts Therapies Research Center, School of Creative Art Therapies, Faculty of Social Welfare and Health Sciences, University of Haifa, Haifa, Israel

**Keywords:** child sexual abuse, time perception, memory deficit, trauma, dissociation, life story

## Abstract

Child sexual abuse (CSA) is a worldwide phenomenon with negative outcomes for survivors whose lives and well-being are compromised into adulthood, due to the trauma caused by the abuse. As trauma survivors often report time and space disorientation as well as memory deficit, an attempt was made to further understand these functions in female adults CSA survivors. More specifically, we questioned how they recalled their past; how their past experience interacted with their experience of the present; and how the past abuse affected the way that they viewed the future. A total of 50 adult female CSA survivors’ open-ended life story interviews were analyzed utilizing Thematic Content Analysis. In relation to time perception and memory deficit, three main themes emerged: Adrift in time and space; disintegration of body, mind and identity; and chaos, exhaustion, and confusion. These themes were discussed from the conceptual perspective of “captured in a distorted time,” which is one of the traumagenic constructs of childhood sexual abuse.

## Introduction

The trauma of child sexual abuse (CSA) is a phenomenon which has been studied extensively, particularly in relation to risk factors and long-term physical and emotional consequences. Of the several forms of abuse (e.g., physical; emotional), CSA is considered to be especially traumatic, due to the internal violation of the child’s body by way of oral, anal or genital penetration (DiLillo et al., 2006). Finkelhor and Browne (1985) attempted, three decades ago, to conceptualize CSA based on their clinical experience at that time. They described four traumagenic dynamics – traumatic sexualization, betrayal, powerlessness and stigmatization – which occur together, as part of a process and form the basis of the trauma unique to CSA. Recently, Lev-Wiesel (2015) suggested a somewhat different conceptualization to the CSA trauma that includes five traumagenic constructs: Soul’s homelessness (split between body and mind), captured in time, entrapped in distorted intimacy, betrayal entrapment, and re-enactment. Time perception is one of the central differences between the two conceptual models, acknowledging the importance of time in providing structure to the human experience.

The objective of the current study was to inquire whether the conceptualization of the distorted time perception caused by CSA can be validated empirically. Based on a phenomenological analysis of life story interviews with 50 adult female sexual abuse survivors, the study examined how female survivors of sexual abuse perceive time and recount their life experiences. This specific issue was chosen based on the importance of time perception and future orientation in endorsing quality of life.

### Child Sexual Abuse

Although the definition of CSA varies widely across studies (Senn et al., 2008), an accepted definition is a sexual act between an adult or a minor with a child in which the child is utilized for the sexual satisfaction of the perpetrator (Briere, 1992). Numerous significant studies define CSA based upon the definition originally proposed by Finkelhor and Hotaling (1984) as “either contact or non-contact sexual experiences between a person younger than 18 years of age and an adult or other person at least 5 years older or sexual experiences resulting from coercion regardless of the age of the other person” (Lalor and McElvaney, 2010, p. 162). CSA is a traumatic life experience that the child is developmentally unequipped to comprehend or process (Cook et al., 2017). It is considered a unique and extreme form of trauma due to the internal violation of a child’s body (Lev-Wiesel, 2008). CSA is known to have medical, emotional and psychiatric consequences (Patensco, 2005; Hetzel-Riggin et al., 2007; Cook et al., 2017) with short and/or long-term effects (Schein et al., 2000; Petrak and Hedge, 2002; Murray et al., 2014) such as depression, anxiety, recurring nightmares and inappropriate sexual behavior (Beitchman et al., 1991; Kendall-Tackett et al., 1993; Macdonald et al., 2006), self-injury (Deiter et al., 2000), suicidal tendencies (O’Leary and Gould, 2009), PTSD, ADHD (Rowan and Foy, 1992; Feather and Ronan, 2006; Lev-Wiesel, 2008; Cantón-Cortés and Cantón, 2010), promiscuity, gynecological disorders, gastrointestinal disorders, eating disorders, and addiction abuse (Beitchman et al., 1991; Hetzel-Riggin et al., 2007). Severity level of symptomatology is contingent upon personal and circumstantial variables such as the identity of the perpetrator and relation to the child (familial, or not), the form of the sexual abuse (harassment, exposure to pornography, with or without penetration, etc.), the duration of the abuse (a single incident or an ongoing occurrence), the location of the abuse (inside the home or elsewhere), personality characteristics, and the child’s emotional resources (e.g., Creamer et al., 2005; Rowen, 2006; BenEzer, 2009; Cutajar et al., 2010).

### Perception of Time, Memory and Trauma

Time perception refers to the subjective manner in which a person experiences the passage of time or the perceived duration of events, which can vary significantly between individuals and circumstances (Carstensen, 2006). While physical time is considered to be objective, psychological time is subjective and pliable with current understanding that the experience of time includes distortions and illusions which influence our perception (Eagleman, 2008) Additionally, our memory process are complex and prone to alterations caused by interior and exterior stimuli such as mood, mental state, cognitive abilities and trauma (Barry et al., 2018). Compared to animals’ biological time processing abilities, there are certain aspects of time perception that are exclusive to the human species, namely the ability to conceptualize time (Pollock, 1971). This appears to be a later evolutionary ability, which might explain the fragility of this mechanism, as it can be interrupted relatively easily. Zimbardo and Boyd (1999) describe people as time travelers, by drawing on past memories, experiencing the present, and looking forward to the future – we obtain structure and cohesion that give meaning to the human experience. In his ground-breaking research dedicated to time perception, Zimbardo concluded that our approach to time reflects upon our decisions, judgments and behaviors – major and minor (Zimbardo et al., 1997), thus influencing our lives in an unequivocal manner. Time perception is considered a complex trait at the base of the human experience (Meck, 1996) – we remember and reflect upon our past, experience our present and plan and anticipate our future. Despite the importance of time as defining our experiences, the nature of time perception and the specific mechanism or mechanisms responsible for it remain a mystery (Le Poidevin, 2015). While the perception of time has yet to be linked to a specific sensory system, psychologists and neuroscientists assume that humans have a system or several inter-linked sensory systems that regulate the mechanism of time perception and its effects on individual experience (Droit-Volet and Meck, 2007). Time as a constant is a feature which provides security as well as meaning to human experience; hence, human fascination and obsession with measuring time and acknowledging it through recurrent ceremonies. Studies have shown that the manner in which time perception is incorporated in the individual experience affects their levels of emotional well-being (Vasile, 2015), satisfaction with life (Carstensen, 2006) and predicts, to some extent, professional success, general health and overall happiness (e.g., Carstensen et al., 1999; Kauffman and Husman, 2004). Memory, while intriguing mankind for at least 2,000 years has been a scientific field only in the past century. Psychologists and scientists have tried to capture its’ elusive nature foremost in the laboratory and consecutively in real-life situations (Baddeley, 2002). Memory is intrinsically linked to time perception, an understanding that came about in recent decades with the recognition that there is a link between fields of psychology that exist on a continuum with overlapping areas. This notion is made more intricate when trauma is involved (Caruth, 1995). Research in the past two decades has shown that a history of trauma increases the likelihood of autobiographical memory deficits, effecting the manner in which individuals recall their life experiences (Droit-Volet, 2012; Ono et al., 2016). Trauma experienced during childhood, while memory systems are developing, is believed to negatively alter the development of basic and autobiographic memory skills (Otgaar et al., 2017).

Trauma is considered a factor that may also alter time perception. The effect of trauma on time perception has been studied extensively (Bonaparte, 1940; Terr, 1984, 1988; Sar and Ozturk, 2008) without the process at the base of time misperception being clarified in full. Initially, time is perceived in an altered manner during the traumatic event (with survivors describing time as slowing down or speeding up), yet this alteration is evident in the aftermath, with survivors relating the traumatic event, as well as subsequent experiences, in a haphazard, confused manner (Terr, 1984). CSA survivors and other trauma survivors often re-experience the trauma of abuse mentally as well as physically when encountering triggers associated with the past traumatic events, a cycle that leads to re-traumatization (Carlson and Ruzek, 2005). The re-traumatization produces despair, due to survivors’ feeling of being trapped in their past and experiencing life through a distorted prism of the past abuse (Lev-Wiesel, 2015). A victim of CSA is encapsulated in the abuse, anticipating the abuse before it occurs, living through the abusive acts when they occur, and anxiously expecting the abuse to reoccur time after time. Being abused becomes the predominant life experience, causing everyday life to fade into the background, with the memory deficit and loss of time perception becoming routine events. Previous research (e.g., Butler, 1999) suggests that the sense of time is closely related to the development of the sense of self. It is therefore understandable that the younger the victim of sexual abuse is, the more severe the time-dependent outcomes are (Briere and Elliott, 1997).

### Captured in Time

Droit-Volet and Gil (2009) studied the time-emotion paradox, which deals with the effect of emotion on the presumably accurate inner-clock humans have been proven to possess. Our time perception is ultimately influenced by our emotions that are prone to fluctuations caused by inner and outer, positive and negative stimuli. Mowrer’s (1960) conditioning theory, which initially clarified learning procedures and subsequently learned reactions to fearful stimuli (which until then was thought to be instinctive as opposed to conditioned), provides a basis for Lev-Wiesel’s (2015) concept of the time entrapment experienced by CSA survivors. Conditioning involves forming an association between two stimuli resulting in a learned response. Thus, when the sexual abuse becomes routine, the child becomes accustomed to familiar signs leading to abuse, and the pairing of the signs with the abuse triggers reactions based upon prior abusive experiences. When this event becomes habitual, the child learns to identify the signs of the impending occurrence, to recognize the peak of the abusive event, and the signals indicating that the specific traumatic occurrence will soon come to an end. When the perpetrator leaves the scene, physical relief is experienced – a sensation of relaxation due to an experiential distancing from the source of stress (the perpetrator). However, this relaxation does not last long. Anxiety and fear gradually increase due to a decrease of the dissociation mechanisms employed during the event itself, and due to the child’s dreading the commencement of the next inevitable frightening encounter with the abuser. Thus, the child is in a never-ending cycle of dreading the inevitable encounter with the abuser, experiencing the abuse, relief when it temporally ends, and dreading the next abusive encounter. This cycle takes a toll on the physical and mental well-being of the child (Olivera-Figueroa et al., 2015) who despite possible employment of dissociation, is left to suffer intrusive thoughts or memories, flashbacks, and other associated stimuli which overwhelm the survivor, throughout her life and do not let her leave the past behind (Panter-Brick et al., 2015). Craig (2009) researched brain functions, providing an explanation for the subjective manner in which time is perceived, with an emphasis on the roles of positive experiences (which cause time to seemingly contract, or speed up), versus negative experiences (which cause time to seemingly dilate, or slow down). The trauma of CSA creates a psychological construct of being entrapped in time, trapped in the past event which feels like the present. This ultimately leads to disorientation and creates a misperception of time (Drake et al., 2008).

## Materials and Methods

The study followed a qualitative research approach, involving the use of the life history interview as the primary method (Bertaux, 1981). This approach puts an emphasis on the communicative elements of storytelling, allowing the participants to choose and accentuate the life experiences at their discretion. It involves a thematic examination of the perceptions and experiences of adult CSA survivors. In addition, this study made use of the qualitative phenomenological method, which allows researchers to encapsulate the subjective meaning of a phenomenon while maintaining the participants’ fundamental perspectives (Moustakas, 1994). In this manner, researchers may present a series of subjective experiences as being part of a larger interconnected structure that can be the foundation of a qualitative-based theoretical model (Strauss and Corbin, 1998). Thematic results were analyzed twice – initially an open analysis was conducted without taking time perception into consideration, in order to discover whether it would naturally and spontaneously emerge. The second analysis was conducted having time perception in mind and looking to find out how participants relate to time and it’s various components, such as perception, memory and quality of life. Narratives were analyzed and successively categorized into themes which provided a basis for the findings.

### Participants and Procedure

Open-ended life story interviews of 50 participants were randomly selected from 300 life story interviews collected by LW since 2010 as part of a broader research. Interviewees’ age ranged between 19 and 64 with the majority aged 30–45. Interviewees represent all familial statuses, with a slight preponderance of married women. Of the women participating in this study, 22 are survivors of interfamilial sexual abuse (by fathers, stepfathers, uncles, cousins, and brothers), 25 are survivors of sexual abuse by an acquaintance (friends, teachers, sports instructors, family friends) and the remaining three were sexually abused by strangers.

Data collection of life-story interviews was carried out by experienced, supervised interviewers throughout 2010–2015, with all participants giving written informed consent to being interviewed and to future use of the data in research. Ethical issues were addressed with an emphasis on the issues of participant confidentiality and dignity. The study was approved by the Haifa University Ethics Committee (#122/15). Participants were given full control over the data collated from them and were offered a referral to resources should their participation cause them duress. Individuals who were diagnosed with psychiatric illness were excluded from the study.

### Data Analysis

A qualitative approach to data analysis was used (Creswell, 1998): The life history interview (Bertaux, 1981), which allows participants to impart their subjective life experience in their words, emphasizing or omitting at their own will (Berntsen et al., 2011). Principles of narrative analysis (Tuval-Mashiach and Spector-Marsel, 2010) were used in order to collect and analyze traumatic events and themes. The life history interview has been used previously in researching the experiences of CSA survivors and found to be appropriate, as the interviewer refrains from sanitizing and distancing the traumatic experience, thus portraying it in an authentic manner (Gonzalez-Lopez, 2015).

The qualitative analysis of the interviews was conducted through Thematic Content Analysis (TCA) (Smith et al., 1992). This analysis aims to acknowledge the basic components of experience, gather them into relevant, meaningful sequences, and then encode and conceptualize the sequences into unique theoretical categories. The next stage of TCA consists of combining a number of theoretical categories into general comprehensive themes.

The qualitative life story approach, chosen for this study, has been validated and found reliable in previous studies (Baerger and McAdams, 1999; Lincoln, 2010; Locke and Lloyd-Sherlock, 2011). Trustworthiness of the study was attained by adhering to measures of reliability. In qualitative methodology this refers to the stability of the results produced, i.e., repetition of the results (Miller, 1986; Smith, 2007).

### Findings

Theoretical categories detected in the data were condensed into general, comprehensive themes, with three time-related themes emerging: Adrift in time and space; disintegration of body, mind and identity; chaos, exhaustion, and confusion. Only the first theme – Adrift in time and space – will be fully presented in this article due to the subject’s scope. Note that participants’ names mentioned in the study are false.

#### Adrift in Time and Space

Child sexual abuse survivors frequently described their lives as characterized by a state of limbo – adrift in space and time without an anchor, devoid of positive memories of the past, prior to the abuse, as the abuse had obliterated their previous identity. (“Memory deficit” is dealt with in the next section.) The interviews made reference to feeling a lack of control over time and confusion at the passing of time. One of the most poignant references to the manner in which being sexually abused impacted time perception was made by Mia, who had been molested by her father from infancy (her earliest memories include being abused) until age 16. When reflecting upon her childhood, Mia summarized her experience as being a prisoner trapped in time:

I was trapped in the past and the present without a future. I couldn’t imagine a future for myself.

Mia’s experience of being imprisoned with no prospect of escape embodies the ongoing terror and trepidation felt by survivors when time does not fulfill its natural role of providing order and direction. These feelings are predominant even years after the physical abuse has stopped.

Another participant, Violet, had been molested at approximately age 11, by her swimming instructor, who also had been a close friend of her parents. Violet described the years after being abused as being a bystander in her own life:

In fact, I didn’t actually live life, I watched life pass by.

While some survivors spoke about time in its general sense, Nancy, who was raped by a friend at age 14, described the time distortion she experienced during and immediately following the rape.

Afterwards, I could barely walk, I couldn’t even think of walking. don’t know how long it took me to climb those stairs, but I did, in excruciating pain. I knocked on the door but no one answered, so I waited for my friend or for someone else, I don’t know for how long. But no one came. After, I’m not sure how long it was, I don’t remember, I returned home to my parents.

Nancy’s frequent referral to her futile attempt to measure time (in this short paragraph she repeated the phrase, “how long,” three times) and her lack of perception regarding time and distance (with 14 steps feeling like an insurmountable hurdle) suggest that, following the rape, she suffered a loss of control over time and space, drifting without the ability to grasp her own experiences in full.

#### Memory Deficit

Survivors’ life stories illustrated the manner in which memories were obliterated by the abuse, with the abusive experience and its constant reenactment in the survivors’ mind leaving little room for other memories. In this manner the abuse obliterates the previous identity, leaving survivors’ non-abusive, healthy past experiences absent from their own lives. This differs from dissociation in its encompassing aspect – memories from long before and after the abuse were demolished, leaving a frustrating blank. The recurring experiences voiced by survivors of memory deficits took a variety of forms. While there were survivors who described being trapped in the past, and others described living parallel lives, Sarah, who had been abused by her father from age six until age 14, describes a lack of connection to her past, an absence of childhood memories:

I don’t have lots of childhood memories. I have lots of flashbacks and lots of black lines. It’s as if I don’t want to remember.

While many people regardless of trauma struggle to recall childhood memories, Sarah experienced the memory lapses in a negative manner – as time lost, black voids in her life which she elusively and unsuccessfully tried to fill. Memories matter because they form a link to the past, acting as an anchor to the present and the future (Terr, 1988). An absence of memories, an elimination of our past experiences, indicates a rupture from our base, without which we exist in a state of limbo.

Similar sentiments were divulged by Victoria, who was abused by her father from age five until she turned 18, when she left her parents’ home upon getting married:

For many years I didn’t remember anything about my childhood, I mean all the years until age five, I simply didn’t remember anything, everything was erased except for flashes, in fact, I only remember myself from age seven.

Both women mentioned negative flashes of memories as opposed to unimpaired memories of experiences, without mentioning positive childhood experiences. Even while the abuse went on, Victoria recalls being ambivalent in relating to it, sometimes believing that she was imagining it:

When we napped, my fathers’ hands used to travel all over, in hidden places. I always felt a sort of doubt. I wasn’t sure what was happening. Did it happen? I used to jump out of the bed and try and check what was going on behind my back. When I thought that my father was rubbing his penis behind me, I was sure that I was hallucinating, that I was insane.

Victoria’s story illustrates the elusive connection CSA sufferers may have to reality. In a world where trauma is rife, right and wrong are reversed. Basic experiences that attach to time and space are muddled, and the result is a confusing void (Sar and Ozturk, 2008).

Caroline revealed the story of her abuse by an artist in a gallery that she had visited with her parents. The artist had offered to paint her picture and then used her parents’ absence to rape her. This occurred when Caroline was 10 years old and subsequently activated a loss of memory spanning the years before and after the rape:

I have no memories of primary school, I know that I was a very good girl, a tidy organized pupil, I didn’t get into trouble. I hardly remember anything from my childhood, most of my memories are things that I was told.

It was not only her childhood memories that were affected, but her ability to perceive time and attach events to a timeline. Caroline was candid about the confusion she lives in regarding her perception of time and experiences:

There are a lot of things that I don’t remember, dates, years, I’m confused about when things happened, when I had a certain job, things like that.

Alice, who had been raped at age 14 and subsequently became addicted to drugs and adapted promiscuous behavior, which led to two abortions before she turned 16, referred to an obliteration of childhood memories from the period before her rape:

I have no special or good memories from my childhood. I only have a few images from my childhood. I don’t have any stable memories.

Once again, images or flashes replace constant, stable memories. Sexual abuse functions as a knife, disconnecting the past from the present and the future. At a later point, Alice chose to refer to her lack of memory once again:

I don’t have any memories. I experienced the world in a fog, like if you pour water on a painting that hasn’t dried yet.

This memory deficit is especially interesting because Alice chose to describe her life as normal before being raped at age 14:

The rape is where everything began. That is where I started deteriorating. My insanity began before the drugs. I dropped out of school straight after the rape. I started working, 12 hours a day, after work I partied and drank, then back to work.

Being sexually abused influenced not only the present, at the time of the abuse, but also the future and, surprisingly, even the past, before the abuse began with a memory deficit a common thread among survivors.

Such is Nancy’s case. Nancy, mentioned earlier, who had been raped by a friend when she was 14 years old, began her interview by saying:

I’ll start by telling about my childhood which I don’t remember much of.

#### Where Was I?

Survivors related to a feeling of missing out on key life experiences and periods following the abuse. The feeling described is more subtle than the memory deficit previously mentioned, varying from a feeling of living on the sidelines to an understanding of time passing without being able to account for their experiences during this passage of time. This lack of knowledge regarding one’s self left survivors struggling with their ability to trust themselves and learn from their experiences. The periods of confusion deny survivors an opportunity to create a cohesive, continuous life story built layer upon layer in an organized manner.

Tammy, who had been abused by her father during puberty, after having been orphaned by her mother, talked about her experiences as an adult that were affected by the abuse. When describing living overseas with her husband and children for two years, Tammy declared:

On the one hand it was an amazing experience but on the other hand, I don’t remember it. I mean that I remember and I don’t remember. I was there or I wasn’t there. The level of emotional involvement wasn’t there, the excitement, the enthusiasm. I remember traveling around Hong Kong, America, seeing amazing sights, but not really. I remember and I don’t remember. There is no absorption. The gulf between the body and the mind is enormous.

The inability to account for their lives leaves survivors in a state of limbo with central parts of their selves a mystery. This may cause anxiety induced by the fact that survivors fear being abused before the abuse they remember, perhaps by additional abusers. The basic need to create order in one’s experiences is unattainable to many survivors of sexual abuse. This was articulated by Marsha, who was abused by numerous men: initially, by a neighbor, when she was only four years old, and later on, by her childhood boyfriend and his friend, from age 12 to 14. At age 20, she was raped by a relative. Like Tammy, Marsha found it difficult to describe specific life experiences, aware of the fact that she does not have a cohesive recollection of the events:

I’m not sure exactly how to begin my life story, I don’t know exactly what happened, I have flashes of memory that I don’t know if they’re true or not. My memories aren’t in order, they are jumbled. There are things I remember clearly and there are things I don’t, I’ve tried to organize my thought lots of times, to think how it started and what happened after that, how it carried on and what ended it, some sort of order.

Similar to other survivors who were interviewed, Marsha alluded to memories appearing as flashes, lacking cohesion or organization.

Stella, who had been molested by her uncle at age 12, suffered from bulimia immediately following the trauma (eating disorders are common following CSA). Stella related a lack of connection in relation to suffering from bulimia:

I guess it’s because of the abuse, when I was about 12 years old I developed an eating disorder. Bulimia. I don’t even remember how it started. I have a sort of blackout. It went on for a long time. Some of it I remember and some of it I don’t.

As stated, confused recollection was commonplace among the survivors interviewed. Most were aware of the fact, often apologizing for their malfunctioning memory, but at times they did not relate the confusion to being sexually abused. Others associated confusion to the abuse, but despite dealing with the issue on a daily basis did not seem to be aware that treatment addressing the issue directly could improve their quality of life. Several survivors were ambivalent about the memory lapses and confusion. On the one hand, they understood that the memory deficit and confusion served to protect them, but on the other hand, they felt anxious and were curious about experiences that they had undergone but could not recollect. Michelle, who had been raped by two men, coherently explained this:

It’s as if my life story has a story inside it that is an unknown, a question mark in my life story that I don’t know.

Being abused is a pivotal life experience; not remembering the abuse means not remembering basic components of one’s identity. The conflict between wanting to remember, being afraid of remembering and having to deal with the inability to remember is at the base of the sexual abuse survivors’ experience.

#### Chained to Trauma

Sexual abuse is a life experience that leaves a residue on survivors’ psyches. The experience cannot be erased or forgotten at will and survivors find their way, often subconsciously, to deal with the negative effect abuse has on them by compartmentalizing it in a manner that allows them to function. They are constantly chained to the traumatic events against their will, much like Prometheus in Greek mythology – Prometheus was a titan who defied the gods and was punished with eternal torment. The immortal Prometheus was bound to a rock, visited each day by an eagle which fed on his liver (considered the source of human emotions in ancient Greece), which would grow back overnight only to be eaten again the following day.

Survivors differed in their coping mechanism following CSA – while some experienced the abuse as a part of their daily lives even after it had ceased, others attempted to obliterate any memory of the abuse by trying to ignore it at will. This being said, abuse has an effect on survivors, who are constantly aware of the abusive experience, regardless of the amount of time that has passed. Some carry the burden of being abused with them constantly, till they detach from themselves or become numb.

The abuse that one carries around constantly acts as a captor, entrapping survivors in a state of disorientation and confusion, where time is meaningless, and the only constant is the abuse itself, as precisely noted by Violet:

Being abused is like cancer of the soul, it finds its way into little cracks like water and stays there.

Between the ages of four and fourteen, Gabriella was sexually abused by her father and at times by her siblings. She used “storage” as a metaphor for dealing with the difficult memories of abuse:

I don’t have many memories. I have flashbacks, pictures, disorganized images. Everything was kept inside, stored away, only images, images.

Gabriella described the abuse as a burden that she carries with her at all times, slowing her down and keeping her rooted in her past, unable to focus on the present or future. This denies Gabrielle a future that is not colored by the trauma that she endured. Along with the traumatic memories, Gabriella stored negative feelings, such as shame, guilt, and blame, which accompanied the abuse:

I remember, I have another image of me at 10 and my brother at eight and we are having sex. The terrible thing is that I think that I initiated it, I felt so guilty. Maybe I also initiated it with my father? I was such a pretty girl, I was really so pretty. But on the other hand that was all I knew, that was what I was taught.

While some survivors claimed to obtain a sense of control over the memories of being abused, others, like Tammy noted that the abuse, even when stored away, was a constant element in her married life:

I remember that while I was married, for quite a few years, 13 or 15 years, the pictures [of the abuse] accompanied me all the time. On the outside, I was one person and on the inside, I was another.

Unlike Tammy, Caroline who was raped at age 10, remembers an active act of erasing the abuse in order to refrain from dealing with it:

As soon as I left there [the art gallery where Caroline was raped] it was over. I erased it. It never happened, I never mentioned it, nobody asked me about it. I never related to it, for many years it was not an issue, it’s as if it never happened.

At a later stage in the interview, Caroline elaborated:

I didn’t actually think about it [the rape] as being abused. I didn’t feel that I was walking around with this detail that I wasn’t telling. It simply didn’t exist. Today it amazes me, the ability that a child has to tell herself a different story, something that allows you to be a child that functions.

Caroline relates to time breaking into two separate entities, a time before the abuse and a time after abuse, a dissociative mechanism which is common among CSA survivors which effects memory and time perception. This rupture impairs the ability to function as a time traveler – having a cohesive bond between past, present, and future – which is at the basis of the normative human experience. A coherent life story fosters a sense of control over one’s life and perception of time and place; a continuous life story means that there is a consistent flow between experiences, knowledge, and sense of being.

While some women carry the abuse with them constantly, alert to the dangers that lurk, others live in a state of denial, not even naming the abuse as such. Both mechanisms are mentioned frequently by CSA survivors.

Some survivors relinquish their story, others refute it; some, like Lisa who was abused throughout her childhood by her grandfather (who she later discovered had abused her mother and additional female relatives) as well as by a male babysitter when she was seven years old, chose to acknowledge her haphazard narration of her life story, storing it within her and revisiting it occasionally:

I’ve told my story so many times throughout my life and it’s interesting that each time I tell it, something new comes up, depending on where I am in life. It’s sad because now I understand that it will always accompany me, I always have a wish that it wouldn’t be so.

Lisa described a lack of continuity in her life story: her personal experiences are ever-changing, leaving her unable to rely on her own experience and knowledge, unable to draw from her past. In a similar manner, Michelle who was mentioned earlier expressed remorse at the extent to which the sexual attack occupies in her life story:

It’s a shame that it happened and it’s such a big part of my life story that I didn’t tell anyone about and it had a huge impact on my life, a bigger impact than I once thought. I feel that it is a part, a large part of my life story and I feel uncomfortable because I want to continue the story and say that there are many other parts to my story, but it does color my story, it affects it.

Michelle lamented over the lack of continuity in her life story, as the abuse had created a break in it. Hearing survivors it is clear – abuse is stored in the body, the mind, the soul – be it consciously or unconsciously. The storing of abuse takes its toll, for it occupies a space within the survivors’ psyches, space which is sometimes akin to a shadow and sometimes to a glaring light. Nonetheless, the abuse is constantly there, a burden carried around and never laid to rest. The past (the abusive experience) takes up a larger part than the actual period in spanned when compared to the present and the future – survivors are imprisoned in the abuse, spending energy and time in an attempt to make sense of the warped time perception and memory deficit it entails in their day-to-day existence.

#### The Blurred Duration of Abuse

The ability to recollect significant life events and to know when they occurred enables one to create a clear life narrative. An additional prerequisite is the need to experience complete occurrences, ones that include a beginning and an end. Survivors’ life stories demonstrated how the invasive nature of sexual abuse colored their experiences and distorted their perception. The abuse became a central feature of their lives, while other activities were pushed aside; the survivors did not have the ability to obtain closure about the abuse or to forge new beginnings. Many survivors described experiencing their lives on parallel lines, one being the actual life, and the other was the abuse and the time and energy spent concealing the abuse. The separation between these parallel, unconnected existences made it difficult to experience time going by in a cohesive manner. This was especially noted with regard to the abuse that had gone on for years. One survivor, who expressed her feeling of shock vis-à-vis the duration of the abuse and the effect it had on the memory deficit characterizing this period of time is Arianna, who had been abused by her stepfather from age nine to 16:

I don’t remember how everything began. I try but I can’t. I have whole years of my life that are erased. Between ages nine to sixteen something important happened but I can’t remember. One day I will, ages nine to sixteen!

In a similar manner, Vera related to the time she kept the secret, after being molested by her uncle, when she was 17. After finding out that her younger and older sisters had both been molested by him, they confided in their father but refrained from telling their mother (whose sister was married to the abuser). Vera drifted through time, without an anchor to ground her. The absence of an anchor means that there is no basis to return to and time loses its key role in providing order:

Looking back it seems absurd to have kept the secret from my mother for so long. How long was it?! I think it was about 10 years. When did it happen?! I remember that I discussed it with my father, I’m not sure exactly when. I know I’m probably contradicting myself with the timeline, never mind.

While telling her life story, Vera was aware of her lack of consistency, regarding her perception of time for the duration of the ongoing abuse. A similar experience was shared by Emily, who was regularly abused by a neighbor, between ages 13 and 18. Emily related to the discrepancy between the ongoing abuse, which occurred at least twice a week for five years, and her lack of comprehension in grasping the extent of the abuse:

When it happens, again and again, you say to yourself, okay, because you have two or three days between each time. Suddenly when you say it went on for so many years, so much time, and I did nothing, I didn’t fight.

A parallel thought was relayed by Nora who was abused as a child by her father, a prominent religious leader of their congregation. When referring to the duration of the abuse, Nora related to the cessation of the abuse as being clouded in her memory:

It went on from the age of eight, until hmmm, I’m not really sure when it ended. I don’t remember the end, I was always afraid that it wouldn’t end, but it was around age 16, 15. It was always a threat. I don’t remember when the actual endpoint was.

Violet, mentioned earlier, who had been molested by her swimming instructor, referred to an objective indicator, the weather, in order to pinpoint when the abuse took place; nevertheless, she was still not sure of her age at the time:

The abuse happened when I was 11 or 12, I was on a swimming team and the instructor messed with me. I don’t remember a lot of the details. I remember that in the beginning, we swam in an outdoor pool and later on when it became cold we swam in an indoor pool, so I know it happened at some point between summer and winter.

While the mechanism of a somatic marker to trigger a memory is a tool utilized regardless of trauma, Violet’s attempt to provide herself with answers about the period of time when she was abused characterizes the frustration mentioned by several survivors who crave knowing exactly what happened, while fearing that discovering information about the trauma they underwent might be too difficult.

Survivors of CSA are frequently abused by numerous perpetrators, sometimes simultaneously and sometimes later in life. This is due to their being groomed by the initial abuser, which makes them vulnerable to additional abusers. Another symptom following CSA is the acquired disposition to choose abusive partners later, as adults, reenacting the submissive role absorbed in childhood. Such is the case of Grace, who was sexually abused by her uncle, beginning at age four. This abuse went on throughout her entire childhood and puberty, with her abuser waiting until she turned 18 to rape her. Grace was subsequently raped by a date at age 21 and again by an acquaintance at age 29. Grace, like other survivors interviewed, could not account for the duration or extent of ongoing abuse:

It started when I was four and carried on while I was in school, I don’t know exactly for how long or how many times. After the third rape I kept asking myself why I suffer from this illness, why it happens to me again and again, what did I do in life to have all these things happen to me.

A warped time perception did not only affect survivors’ waking moments, but their sleep, as well. Abigail, who was sexually abused by her brother for a decade, from age seven, and sexually abused by a family friend from age 14, spoke about the distorted sleeping patterns she still suffers from, as an adult, a decade after the abuse ended:

Part of the problem is that it stays with you, not the actual rape, but the sleeping pattern. Even today when I get into bed I automatically think – maybe tonight the abuse will end – I try and figure out how many hours until the abuse begins and how long I need to stay awake and how many stories I need to tell myself until it ends. These are the remnants of the abuse. I am still trying to get rid of them.

Abigail explains that once she understood that the abuse was not normal behavior, her sleep, during the years of abuse, was disrupted:

It took me a few years to understand what was going on, after all, I was a young girl, it was only at 14 that I understood that it was wrong. I stopped sleeping, I didn’t fall asleep for 5 days and then I collapsed for a whole day, and so on. When I was 15 I started seeing a psychologist and a psychiatrist, taking medication, to sleep, for depression, for anxiety, for a million different things.

As demonstrated in these narratives, abuse impacts the ability to experience time in an orderly manner; sleep patterns are disorganized; and even the capacity to gauge the extent of time the abuse went on for is disturbed. The blurred duration of abuse, lacking a definitive beginning and more essentially an absolute end, create a situation in which CSA survivors are stuck in the drama of abuse, unable to partake in real-life experiences, often failing to keep up with their peers in age-related milestones such as academic studies, marriage, children, careers. This confusion regarding actual life as opposed to a virtual existence is at the core of the traumatic existence many survivors experience throughout life.

#### Dissociation as a Coping Mechanism

One of the functions that time fulfills is that of putting experiences into a context, enabling a creation of order: for the past, it is remembering experiences and linking them to outer stimuli; for the present, it is relating to ongoing experiences; and in the future, it is about planning ahead and anticipating. Time is an anchor in the sense that it is inflexible, not governed by human whim, not pliable or open to manipulation. These qualities ensure that time is a constant, a known factor, a reliable moor in a sea of unknowns that make up life. When the ability to perceive time is affected, the imperative ability to rely on time is disturbed.

Survivors related to a central factor that caused them to feel adrift – dissociation – during the abuse or following it. The common thread is the sense of loss: loss of connection to reality, loss of time, loss of an anchor to ground them. This loss leaves survivors adrift in an abyss where time and space are deflated.

Dissociation is a detachment from reality, a term that includes an array of behaviors on a continuum – from mild detachment, which occurs while daydreaming, to severe pathological detachment, which can include amnesia, loss of identity and fragmentation of identity. As dissociation is a coping mechanism, aimed at minimizing stressful situations, it is not surprising that countless studies have shown a correlation between trauma and dissociative tendencies, with sexual abuse figuring recurrently as the ground upon which dissociation flourishes.

The majority of survivors who partook in this study commented about experiencing dissociation, or referred to dissociative experiences that occurred during the abusive encounters or in the aftermath of being abused.

Sarah, mentioned previously who was abused regularly by her father, depicted the dissociative experience in almost whimsical terms that are in stark contrast to the horrific experiences that she endured:

The experience was so difficult that I disconnected. My body went through what it went through while my soul was sitting up on a cloud, swinging and watching until it was too difficult.

Sarah related to dissociation as an experience of being split between the physical and the metaphysical, between the traumatic abuse and the outer-worldly, an experience of being disconnected from time and space.

Bella, who had been molested at age eight by a teenager who lived in her neighborhood, was acutely aware of her inclination to dissociation: *Basically, what I understood with time is that from age eight to about age 18 I was in a state of disassociation, not completely but for a large part of the time I was disconnected on an emotional level. It’s as if while I’m talking to you I’m experiencing the conversation from the side. I’m constantly seeing and critiquing myself, even while I’m talking to you I’m having a conversation with myself at the same time. When dissociation happened I would have an ongoing dialogue with myself, criticizing myself – what are you doing? What are you saying? How are you dressed? What are you doing here?*

Despite acknowledging her dissociation by name, Bella downplayed its effect on her life:

My dissociation was minor. It didn’t affect my day-to-day functioning. I wasn’t a disconnected, wounded child.

Bella attributes her difficulties with relationships with men and her choice of a same-sex partner to being abused but talks about all of this in a detached manner:

As far as relationships and intimacy go I don’t feel that it [the molestation] affected me. Apart from the fact that I’m in a relationship with a woman [laughs]. Maybe it does have something to do with that. I am attracted to men, I’ve had a relationship with a man, my first love when I was 16. After that, I had 2 relationships with women and then different interactions with men. I feel that with men I am attracted to them but there is a sort of blockage on an emotional level that stops me from getting close to them, I was disconnected, I felt as if I was acting the situation not actually experiencing it.

Bella even described initiating an encounter with her abuser, when she was an adult. The encounter was a reenactment of her childhood trauma, during which she dissociated, just as she did as a child:

I went over to his house and there was a situation, a sexual attack, not a violent one, it was something consensual. Until I started therapy I didn’t remember it at all. I remembered how I got there, at the beginning of the conversation, I remembered up to a point and in fact, I didn’t remember anything. I think that is one of the things that protected me.

Along with the dangerous behaviors, Bella described the loneliness that accompanies dissociative episodes:

Even when I was in a relationship I felt lonely. As if there is still a part of me that isn’t connected, that’s disconnected from the two of us as a couple, as if there is something that isn’t joined.

Like Bella, Nancy who was raped by a friend from her neighborhood when she was 14, referred to her dissociative tendencies as confusing and dangerous:

All this disconnectedness, there is so much of it, I can be having a conversation with a friend and decide that I want to have a shower and invite them to shower with me so we can continue talking, without understanding that inviting a guy to shower usually leads to something. I completely separate the intellectual level from the physical level, I’m completely clueless about what is happening.

Nancy was extremely confused in the recounting of her life story; she lost her train of thought repeatedly, and she shifted between different periods of time that she experienced in a seemingly random manner, she described feeling disengaged from her surroundings:

Many times I feel as if I’m behind a glass window like in a radio show, and I can scream but nobody will hear, nobody notices me, nobody sees my distress.

Emily, who was introduced earlier had been repeatedly abused by a neighbor from age 13, she also described her dissociation in exact terms:

I disconnected my body from my feelings. I disowned my body. I gave my body away.

Susan, who had been molested at age eight, by the pool cleaner, also referred to dissociation specifically:

Even today when I try to remember what happened, who I told first from my family, I cannot remember, it’s like I have a blackout about what happened inside that bathroom. I usually remember things in detail, but about this situation I have lots of blackouts, I use this dissociation to deal with difficult situations in my life.

Susan mentioned dissociation as a coping mechanism that commenced when relating to the abuse but continued to accompany her throughout life. A similar coping technique was used by Nadine, who was raped by her uncle during a family trip at age 11 and abused repeatedly by her father, for six years from age, 11 until age 17:

To escape the physical pain I taught myself to close my eyes and go inside to block out the pain. Even now, after all this time I still can’t go into my own mind to see all the details of what happened that day.

Ruth, who was molested by a teenage neighbor when she was six years old, experienced “seeing herself from the side” during the abuse. The dissociative episodes continued, years after the abuse had ended, as Ruth relates:

I remember that he used to lay me down on the bed and I used to see it from the side […] in high school and in university, I was in a different world, I could go to different places in my mind as if I was not in the class. I used to think about different things or daydream and imagine things.

Caroline, whose case was introduced earlier, also described seeing the abuse from outside herself:

We visited a gallery and the artist told my parents that I was a pretty girl and he offered to paint me, he told them to leave and come back and he started painting me and moving closer and closer until he was very close, our knees were touching, and at some stage he was without his pants, his penis was between my legs, and his hands were all over…and I don’t remember what he looked like, I only remember the physical pain and I remember seeing everything from above, as if my soul was floating above and I remember thinking how I could get out the door, I don’t remember anything else, I remember thinking that it is as if this wasn’t happening to me, I remember thinking that the door was so close but not understanding why I couldn’t get up and reach the door, I remember that he carried on painting and he opened the door and at some point my parents came, I know that they came because I remember arriving at home and the painting hung in the house. I don’t remember, I don’t remember the details, I don’t remember that thing at all.

Abigail, previously mentioned, was sexually abused by her older brother and a family friend for a decade, from age seven Abigail described an attempt to dissociate by feigning sleep:

During those 10 years, I acted as if I was sleeping every night, every night one of them came to me and did what they wanted and I simply didn’t respond. I believed that as long as I act as if I’m sleeping, maybe they won’t know that I know.

At a later point during the interview Abigail referred to dissociating by detaching her nighttime abusive experiences from her daily life:

For many years I completely disconnected what went on in the day from what went on in the night.

Some of the survivors interviewed, such as Ariana, who was abused by her stepfather from age nine to age 16, described dissociative experiences without utilizing the term “dissociation”:

I have always known that there is secrecy regarding sex in my past, but I denied those thoughts because of the pain and the shame that this caused me, thoughts pop into my mind, without being put to words.

Evelyn was abused by numerous men, from age five to 15; those she remembers include her father, stepfather, step-grandfather and other caregivers. She described her dissociation akin to the experience of fainting:

I do not remember, it is as if I fainted and have no recollection […] remembering is horrible but not being able to remember is even worse.

Evelyn spoke of what can be described as the drawback of dissociation, the inability to process the abuse in its entirety. This being said, the dissociative mechanism is ultimately one which protects survivors.

Dissociation begins as an automatic coping mechanism, aimed at protecting the survivor of abuse from having to deal with the horror of being abused, but often has devastating consequences. For example, dissociation can lead to a false sense of control that shatters when triggered by experiences outside the survivor’s control, or when the survivor feels capable of dealing with the memories of abuse but is unable to recall them. Dissociation enables escape into another space and time, detachment from the body being abused. This disintegration of actively partaking in life is parallel to the disintegration of body and soul bought upon by the abuse.

Miriam, who was raped at age 17 by a close friend, described carrying on with her life in what she perceived to be a normative manner:

I convinced myself that I was okay. It’s easy to live your life when you convince yourself that this is your life and you deal with the situations that arise. When you convince yourself, you start to believe it. That was my mistake. During my studies, there was a visit to a jail and I saw him there. I only glanced at him but I ran out and that is when the flashbacks began – nightmares, crying fits, sleepless nights, anxiety – from there the path to alcohol was very short. I stopped functioning and couldn’t drag myself out of bed. I became suicidal.

Through dissociative mechanisms, Miriam felt falsely protected. When dissociation ceased to function, she was abruptly confronted with the past in a way that felt overwhelming, leaving her distraught and helpless. Miriam, who spiraled down into self-destructive behaviors, pinpointed the dissociation from her body to the rape itself:

I got into bed with men and after heating them up I’d run away. If I did sleep with someone, after one time I’d leave him. It was a kind of revenge. It’s like being a prostitute but not really, it’s more about revenge. You don’t actually feel that your body is yours, you don’t feel any connection to your body, it’s only a tool to move around as if the guy who raped me took it away from me.

In the same way, Tammy, formerly mentioned, described an exterior existence in which she functioned normally, while actually being disconnected:

After the abuse stopped I was very busy and active at school and in the youth movement, but I was actually cruising through the days without knowing where I was, which world I was in. Now I understand more how disconnected and absent I was.

Some CSA survivors did not mention disconnecting during the abusive acts but referred to later dissociative experiences, often linked to consensual sexual relations. Such is the case of Nora who was presented formerly – Nora’s father, a religious figure in their community abused her and her sister as children. Nora described the sexual relations with her husband following their marriage in the following manner:

We didn’t manage the first night, but I had a good experience. From him, I felt respect, love, gentleness, listening, he was very loving and embracing. I was sort of paralyzed. Not physically paralyzed, I was there, I remember it. I was active. I was part of it. No one did anything that I didn’t want, not at all, but there was a sort of shock. At a certain level, a very minor level there was a disconnection between me and what was happening.

Dissociation is an important mechanism that allows the traumatized CSA victim to protect herself and continue with what, from the outside, can be construed as normative behavior. However, delayed dissociation can be alarming if it ends abruptly, without having alternative coping mechanisms in place. Dissociation in itself is one of the factors that contribute to a misperception of time – experiences are slowed down, sped up or completely erased (on a conscious level), leaving survivors with gaps in their life story. As demonstrated, these gaps range from minutes to years, with a wide range of disparity.

## Discussion

### The Sisyphean Existence of CSA Survivors

Survivors who partook in the study were not asked directly about the subject of time perception but nonetheless related to the subject repeatedly. References to time and time perception were spontaneous and prevalent, with participants often relating to time in a wistful and melancholic manner, at times enraged at the passing of time, in a way that they found unsuited to their experience (feeling that time was fleeting or ceaseless) and sometimes describing time as a captor or restraint, holding them back from progressing in their lives. Survivors referred to attempts to escape their past and detach from the trauma yet, failing to do so, found themselves repeatedly being pulled back by memories, flashbacks, and disorientation.

In Greek mythology Sisyphus is known for endlessly rolling a boulder up a hill, only to have it roll down time and time again, as a punishment bestowed by the gods for cheating death (Raffalovich, 1988). His name has become synonymous with a futile, frustrating and unrewarding task. The life experiences repeatedly described by survivors resemble this myth. Survivors described an uphill battle for their physical and mental well-being, which included three stages: they initially experienced being adrift in time and space followed by disintegration of body, mind, and identity that subsequently led to chaos, exhaustion and frustration. These stages repeated themselves over and over again, in a cycle of entrapment.

The CSA survivors who partook in this study referenced time, time perception and memory deficit repeatedly, when narrating their life story. The recurring mention of time-related issues suggests an ongoing preoccupation with time and its passage and its significant impact on their lives. Being abused summons a disintegration of self (Rowan and Foy, 1992), while it is initially the body that suffers abuse, the mind is drawn into the traumatic experience; feelings such as betrayal, shock, confusion, horror and chaos shadow positive feelings, such as satisfaction, accomplishment, joy, and happiness (Leserman, 2005). The abusive act raises questions regarding one’s identity, as it is thrown into turmoil, upsetting the delicate balance of self. It is upon this disintegrating foundation that CSA survivors are left to fight for a sense of order in their lives. This is one of the stages upon which time misperception occurs – if time is the basis upon which we evaluate and validate our experiences, impairment of this primal ability affects us. The experience of being captured in time, following CSA, as presented by Lev-Wiesel (2015) was evident in the life stories of survivors who participated in this study. A relentless re-experiencing of trauma occurred for years after the abuse had ended, triggering feelings of despair and frustration among survivors who sense that they will never come to terms with their pasts. This colors their present and future, affecting their life experiences in a detrimental manner. While time disruptions caused by tiredness and jet-lag can have relatively minor and short-term effects, traumatic experiences leave a lasting imprint in our psyches, an imprint which tampers, among other things, with our capacity for time perception and autobiographical memory. Due to the intrinsic nature of time perception, which is deep-rooted in us, the damage caused by trauma can often be initially undetectable and consequently difficult to repair, once uncovered. The experience of being trapped in trauma undermines survivors’ struggles to heal, as the traumatic event draws them in repeatedly through intrusive thought, flashbacks and stimuli associations.

From the interviews, it became apparent that survivors perception of time is affected in a similar manner, with survivors repeatedly referring to common experiences relating to their perception of time that were damaged following their abuse; they described a constant disintegration of their minds, bodies, and identities, leaving them adrift in a time and space, without the ability to grasp or order their past experiences or to objectively anticipate a positive future. Survivors who were sexually abused as children are akin to buildings without solid foundations – unable to build additional floors as the delicate, breakable foundation is not strong enough to endure any advancement, leaving the building incomplete and vulnerable, powerless to survive the elements.

## Conclusion

**FIGURE 1:**
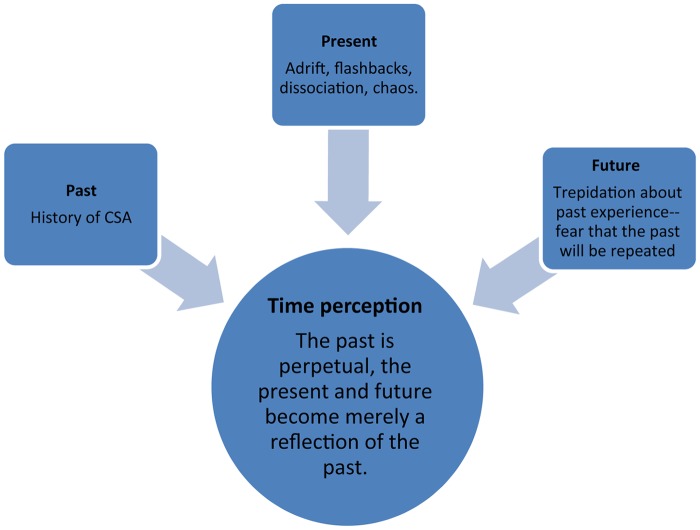
Time perception as affected by CSA.

Based upon the data collated and analyzed, following is a model relating to the effect CSA has upon survivors’ time perception. The model relates to the normative manner in which time is perceived, alongside the warped manner in which it is perceived by CSA survivors. While the majority of people experience the past as a collection of reflections and memories, positive and negative, the past portrayed by CSA survivors was filled with memories of abuse and trauma that obliterated other memories. The present was usually experienced as a chaotic state of limbo rife with flashbacks and dissociative episodes. CSA survivors suffered trepidation and fear that their past will be repeated in the future, without the normal anticipation and planning (see Figure 1).

The warped perception of time described is fertile ground for the feeling experienced by survivors – an uphill, ongoing battle for their wellbeing, which included three stages: a state of limbo adrift in time and space followed, by disintegration of body, mind, and identity and ultimately leading to chaos, exhaustion and frustration. The fact that survivors are trapped in a vicious cycle for years raises the question about the brain function area that might be affected by this traumatic experience. That is, to what extent is CSA trauma registered in the brain and, if so, how could this be treated. A recent study on the revival of dissociated memories as a result of hyperbaric oxygen treatment seems to imply that this kind of treatment might reactivate somatosensory brain areas that were damaged due to CSA (Efrati et al., 2018). Nonetheless, the current study implies that the issue of time perception should definitely be further studied and included within the focal of treatment.

## Author Contributions

RL-V was responsible for data collection. AJ-K conducted thematic data analysis. Both authors jointly completed the discussion, contributed to manuscript revision, and approved the submitted version.

## Conflict of Interest Statement

The authors declare that the research was conducted in the absence of any commercial or financial relationships that could be construed as a potential conflict of interest.
